# Perinatal Stroke and Cerebral Sinovenous Thrombosis Caused by Congenital Nephrotic Syndrome *NPSH1* (Finnish Type): A Case Report

**DOI:** 10.1055/a-2655-9135

**Published:** 2025-07-28

**Authors:** Bregje O. van Oldenmark, Vivianne E.H.J. Wintjens, Menno J.P. Toirkens, Roos W.G. van Rooij-Kouwenhoven, Enrico Lopriore, Linda S. de Vries, Sylke J. Steggerda

**Affiliations:** 1 Division of Neonatology, Department of Paediatrics, Willem-Alexander Children Hospital, Leiden University Medical Center, Leiden, The Netherlands; 2 Department of Radiology, Leiden University Medical Center, Leiden, The Netherlands

**Keywords:** congenital nephrotic syndrome, cerebral sinovenous thrombosis, thalamic hemorrhage

## Abstract

Congenital nephrotic syndrome (CNS) is a severe renal disorder in newborns, characterized by complications such as albuminuria, hypoalbuminemia, and hypercoagulability. While CNS is known to predispose patients to thrombosis over time, to our knowledge, cerebrovascular complications such as cerebral sinovenous thrombosis (CSVT) within the first week after birth have rarely been reported before in neonates with confirmed CNS. We present here an infant, born by normal vaginal delivery, which was complicated by the retention of a large placenta. She was first admitted on day 3 with perioral cyanosis and polycythemia. She developed apneas that were later confirmed with amplitude integrated EEG to be seizures and was found to have multiple thrombotic complications, including extensive CSVT and bilateral thalamic hemorrhages. Serum albumin level was very low, with high urinary levels suspicious for Finnish-type CNS, which was confirmed by *NPHS1* pathogenic variants p.Cys623Phe and p.Asn870Profs*36. Despite partial exchange transfusions and anticoagulation therapy, the infant developed severe cerebral abnormalities. This case underscores the importance of considering CNS in neonates with a large placenta, severe polycythemia, proteinuria, and hypoalbuminemia, as they may be at risk of developing CSVT.

## Introduction

Congenital nephrotic syndrome (CNS) is a severe renal disorder that typically presents within the first 3 months after birth, marked by the triad of nephrotic-range proteinuria (>200 mg/mmol creatinine), hypoalbuminemia, and clinically evident edema, and with an incidence ranging from 1 to 3 cases per 100,000 live births. 1 This autosomal recessive condition is often caused by genetic variants that impair the glomerular filtration barrier. Among its subtypes, Finnish-type CNS is the most prevalent, occurring in approximately 1 per 10,000 live births. 2

Finnish-type CNS is caused by pathogenic variants in the *NPHS1* gene, which encodes nephrin, a critical protein of the slit diaphragm in the kidney's filtration barrier. Defective nephrin leads to proteinuria, resulting in albuminuria, hypoalbuminemia, edema, hyperlipidemia, and a hypercoagulable state due to dehydration and urinary loss of antithrombotic proteins. As a result, these disturbances increase the risk of thrombosis, malnutrition, infections, and hypothyroidism. 3

Although CNS is known to lead to a hypercoagulable state over time, cerebrovascular complications such as cerebral thrombosis—arterial or venous— have, to the best of our knowledge, rarely been reported in neonates with confirmed CNS during the neonatal period. Here, we present a case of a neonate who developed extensive cerebral sinovenous thrombosis (CSVT) with bilateral thalamic hemorrhages and severe white matter injury in the context of the Finnish-type CNS.

## Case Description

A female infant was born at 38 weeks' gestation to a 37-year-old Caucasian woman (gravida 3; para 2), with no significant medical history. Pregnancy was complicated by the presence of diabetes gravidarum and suspected fetal macrosomy, requiring treatment with insulin. The infant was born after an uncomplicated induced vaginal delivery, with a birth weight of 3,390 g (p60-70). Apgar scores at 1, 5, and 10 minutes were 9, 10, and 10, respectively. There was retention of the placenta, requiring manual removal. The placenta was notably large (>p90) and was extracted in segments, which were subsequently submitted for histopathological analysis. Laboratory results were within the normal range after birth, and therefore, the infant was discharged home after 2 days.

On postnatal day 3, the infant was admitted with episodes of perioral cyanosis during feeding. Laboratory evaluation revealed polycythemia (venous hemoglobin 25.6 g/dL, hematocrit 71%) for which the infant was treated with two partial exchange transfusions. Additionally, she developed apneic episodes for which continuous positive airway pressure (CPAP) with supplemental oxygen was initiated. Antibiotics were administered due to suspected neonatal sepsis; however, all blood samples and a cerebrospinal fluid sample were negative for infection. On day 5, the infant developed clinical seizures, presenting with apnea and opisthotonos. The infant was transferred to the neonatal intensive care unit (NICU) of our tertiary center for neuromonitoring, which confirmed seizure activity. Phenobarbital was initiated as the primary antiseizure medication; however, due to inadequate seizure control, midazolam was subsequently administered, resulting in cessation of the seizures. Following this intervention, the infant developed respiratory insufficiency, requiring intubation and mechanical ventilation.

**Fig. 1:**
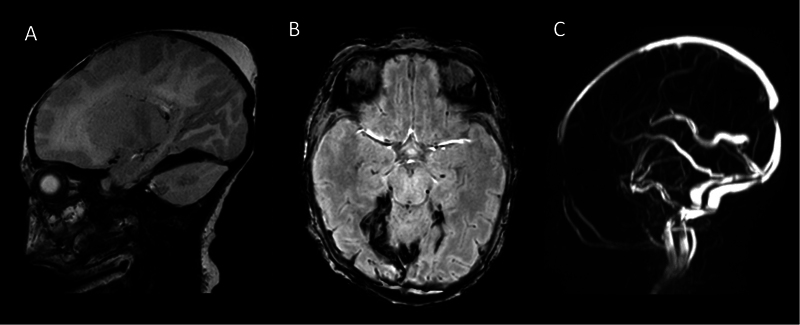
Neuroimaging on admission. MRI, postnatal day 6. ( **A** ) Parasagittal T2-weighted image on the right side showing a small collection of subdural hemorrhage around the tentorium. ( **B** ) Axial susceptibility-weighted image (SWI) showing a small collection of subdural hemorrhage around the tentorium, more prominent on the right than on the left. ( **C** ) Magnetic resonance venography (MRV) demonstrates the patency of both the superficial and deep venous systems. MRI, magnetic resonance imaging.

**Fig. 2:**
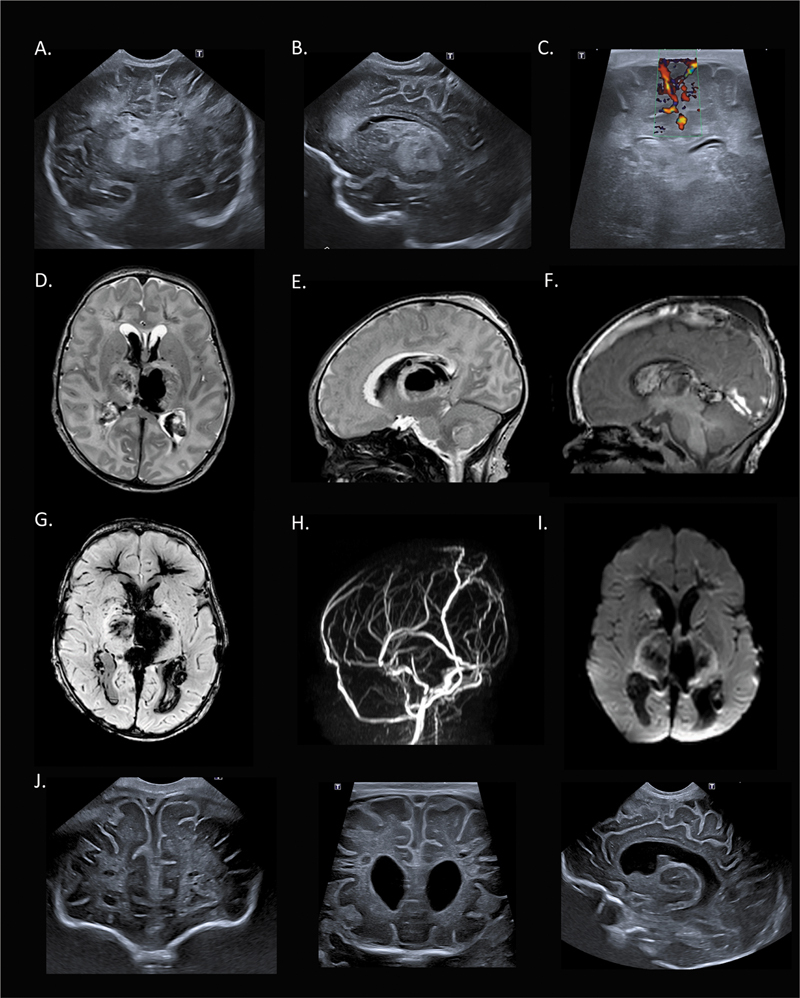
Neuroimaging on postnatal day 10. Upper row: ( **A** ) Cranial ultrasound (cUS) on day 10 in the coronal plane demonstrating bilateral intraventricular hemorrhage (IVH), echogenicity in both thalami suspected of thalamic hemorrhage, and inhomogeneous periventricular white matter abnormalities. ( **B** ) cUS in parasagittal plane demonstrating IVH and thalamic abnormality. ( **C** ) cUS Doppler imaging demonstrating a thrombus and absent flow in the superior sagittal sinus (SSS). Second and third row: Magnetic resonance imaging (MRI) on day 10. ( **D** ) Axial T2-weighted image revealing bilateral thalamic hemorrhages, more pronounced on the left, with bilateral IVH and mild ventricular dilatation. The thalami are hyperintense and swollen. Also note the low signal in the trajectory of the deep medullary veins (venous tree), especially in the frontal lobes. ( **E** ) Parasagittal T2-weighted image on the left side showing IVH and thalamic hemorrhage. ( **F** ) Midsagittal T1-weighted image demonstrating a hyperintense thrombus in the SSS, confluence, and straight sinus ( **G** ) **.** Axial susceptibility-weighted imaging (SWI) confirms IVH and bilateral thalamic hemorrhage, and in addition, shows bilateral hemorrhagic changes in the frontal deep white matter. ( **H** ) Magnetic resonance venography (MRV) depicting absent flow in the SSS, internal cerebral veins (ICVs), inferior sagittal sinus (ISS), straight sinus up to the confluence, ( **I** ) axial diffusion-weighted imaging (DWI) showing diffusion restriction in both posterior limbs of internal capsule (PLIC) and borders of the thalami. Lower row: ( **J** ) cUS at 3 weeks showing bilateral frontoparietal periventricular white matter cysts, progressive ventriculomegaly, and tissue loss in both thalami.

Upon admission to the NICU, cranial ultrasound (cUS) appeared normal, and magnetic resonance imaging (MRI) on day 6 revealed no intraparenchymal abnormalities, except for a small collection of subdural blood adjacent to the tentorium and around the right temporal lobe. Magnetic resonance venography (MRV) showed patent sinuses ( Fig. 1 ). Following stabilization on day 6, poor perfusion of the legs was noticed on day 9, after removal of a femoral artery catheter. Duplex ultrasonography revealed occlusion of the right superficial femoral artery, and anticoagulation therapy with low-molecular-weight heparin was initiated. On day 10, the patient's clinical condition acutely deteriorated with recurrent seizures, treated with phenobarbital, midazolam, and lidocaine. cUS revealed bilateral intraventricular hemorrhage (IVH), bilateral thalamic hemorrhages, and extensive white matter lesions attributable to CSVT involving both the superficial and deep venous systems, as confirmed by absent flow on color Doppler ultrasound. MRI, performed on the same day, demonstrated extensive CSVT involving the superior sagittal sinus (SSS), straight sinus, internal cerebral veins, and proximal transverse sinus. MRI confirmed the bilateral thalamic hemorrhages, as well as venous congestion and hemorrhagic infarction in the periventricular white matter ( Fig. 2 ). Additionally, restricted diffusion in the posterior limb of the internal capsule was visible. Abdominal duplex ultrasound on the same day revealed thrombi in the left subclavian, axillary, brachial, basilic veins, and in the left branch of the portal vein. No abnormalities were noted in the spleen, liver parenchyma, kidneys, or bladder. Coagulation and thrombophilia testing (including antithrombin and Protein S levels) showed values within the reference range for neonatal age.

Additional blood tests showed normal glucose, but extremely low serum albumin (11 g/L; reference range 34–48) and protein levels (29 g/L; reference range 51–73). Subsequent urine analysis on day 10 showed an increased albumin level of 3,300 mg/L and a protein/creatinine ratio >6,600 mg/mmol, which raised—in conjunction with the extensive cerebral thrombosis on neuroimaging—the suspicion for CNS. The infant did not show signs of edema.

Whole-exome sequencing (WES) coupled with Human Phenotype Ontology analysis was conducted. Relevant Human Phenotype Ontology terms included *abnormality of coagulation* (HP:0001928), *seizure* (HP:0001250), and *nephrotic syndrome* (HP:0000100). While awaiting the results of genetic testing, the infant was supported on the NICU with albumin transfusions for circulatory instability. The infant's thyroid function tests revealed a TSH level of 8.3 μIU/mL and an free T4 level of 9.6 pmol/L, prompting treatment with levothyroxine (12.5 μg once daily). A repeat cUS 13 days later showed cystic evolution of the white matter and development of posthemorrhagic ventricular dilatation ( Fig. 2 ). Due to the severe cerebral injury, the infant was considered unsuitable for hemodialysis in infancy and was expected to undergo kidney transplantation. After the withdrawal of life-sustaining medical treatments, the infant passed away on day 25. WES confirmed Finnish-Type CNS, with compound heterozygosity in *NPHS1* , involving a pathogenic missense variant (maternal) and a pathogenic frameshift variant (paternal). In addition, placental pathological examination supported the diagnosis of CNS, revealing clusters of villi with stromal edema and detachment of the trophoblastic basement membrane, consistent with hydropic changes.

## Discussion

To the best of our knowledge, this is one of the first reported cases in the literature of an infant with CNS who developed extensive CSVT and cerebral injury during the neonatal period. The other case was described by Fofah and Roth in 1997, prior to the introduction of genetic testing, such as WES. 4 In contrast to their report, we also show the neuroimaging features and were able to confirm the diagnosis of CNS in our patient with genetic testing. In addition, Horsch et al., reported a term neonate with prenatally diagnosed CNS who presented with bilateral cerebral hemorrhages and a thrombus in the right common carotid artery. However, there was no mention of CSVT. 5

During hospitalization, the infant developed extensive cerebral thrombosis involving both the SSS and deep venous system, accompanied by IVH, thalamic hemorrhages, and severe white matter injury. Additionally, duplex ultrasonography showed thrombi in the femoral artery, the left subclavian, axillary, brachial, and basilic veins, as well as in the left branch of the portal vein.

Several mechanisms underlie the hypercoagulable state observed in the CNS. Glomerular dysfunction leads to significant urinary loss of key antithrombotic proteins, such as antithrombin III and protein S, combined with increased hepatic synthesis of prothrombotic factors. 6 However, routine coagulation study results and thrombophilia testing in our infant were within the normal neonatal ranges and could not explain the extensive thrombosis observed, which progressed dramatically just over 5 days. No other risk factors, such as dehydration or sepsis, were identified.

Cerebrovascular complications in children due to a hypercoagulable state in nephrotic syndrome were first documented by Schwarz and Kohn 7 in 1935. Later, in 1988, Igarashi et al. described cases of cerebrovascular events in infants with pediatric nephrotic syndrome. 8 A recent systematic review identified 62 pediatric cases of CSVT associated with nephrotic syndrome, noting that most occurred within 6 months of disease onset and were more frequent in males. 9 While thromboembolic events are well-documented in children with nephrotic syndrome, most published cases focus on children rather than neonates, and CSVT in the neonatal period remains exceedingly rare.

In this case, our patient carried two previously reported pathogenic *NPHS1* variants in compound heterozygosity: A maternal missense known pathogenic variant (c.1868G > T, p.Cys623Phe) and a paternal novel frameshift variant (c.2606_2607dup, p.Asn870Profs*36), both classified as pathogenic according to the American College of Medical Genetics and Genomics and the Association for Molecular Pathology (ACMG/AMP) guidelines. Pathogenic *NPHS1* variants disrupt the filtration barrier, resulting in massive proteinuria, hypoalbuminemia, and nephrotic syndrome.

Different symptoms in this case raised the suspicion of CNS. Notably, serum albumin levels were extremely low, and additional urine testing revealed severe proteinuria on day 10. These findings, combined with signs of polycythemia and the extensive CSVT, eventually led to the diagnosis of CNS. Severe proteinuria resulted in the loss of immunoglobulins, antithrombotic proteins, and thyroglobulin, with the latter contributing to hypothyroidism. Extrarenal manifestations were minimal, consistent with *NPHS1* -associated CNS, as the *NPHS1* gene is specifically expressed in renal podocytes. Although no histological examination was performed, such cases typically reveal the hallmark feature of irregular microcystic dilatation of the proximal tubules. 10 Regarding placental pathology, findings were in-line with the diagnosis of CNS, particularly the prominent trophoblast and hydropic alterations. An increased placental weight (>25% of birth weight) is also frequently observed in this diagnosis. 11 There were, however, no other abnormalities during pregnancy that could have suggested CNS, nor was the placenta considered abnormal on fetal neuroimaging.

Treatment of CNS is complex and focuses on supporting growth while managing proteinuria and its complications. Venous lines are often necessary for a long time, but these pose significant risks of infection and thrombosis. Chemical nephrectomy, using ACE inhibitors to reduce proteinuria, results in renal insufficiency, necessitating peritoneal dialysis to maintain kidney function until the child becomes eligible for kidney transplantation (typically >10 kg, usually >2 years old). 12 This demanding course imposes substantial physical, emotional, and logistical burdens on the child, family, and health care team. In this case, the presence of extensive thrombosis and severe cerebral injury led the multidisciplinary medical team to deem the described treatment pathway not in the child's best interest.

In addition, WES revealed that both parents are carriers of the pathogenic variant, giving their future children a 25% chance of developing CNS. It is essential to provide genetic counseling to inform them about the associated risks and options for prenatal genetic testing. To ensure appropriate treatment immediately after birth in future cases, diagnosing CNS in utero is preferable. Elevated levels of alpha-fetoprotein (AFP) have been suggested in the literature as a potential prenatal marker for CNS. Since the 1970s, AFP measurement has been used for the prenatal diagnosis of congenital nephrosis. However, as described by Patrakka et al., elevated AFP concentrations and proteinuria in fetal kidneys are observed in both CNS cases and carriers, making AFP-based diagnosis unreliable. The authors strongly recommend analysis of pathogenic variants in the *NPHS1* gene to confirm a prenatal diagnosis of CNS. 13

In conclusion, this report shows that in case of an infant with an enlarged placenta at birth, severe polycythemia, hypoalbuminemia, and thrombotic complications (including CSVT), CNS should be included in the differential diagnosis.

## References

[1] Arbeitsgemeinschaft für Paediatrische Nephrologie Study Group Hinkes B G Mucha B Vlangos C N Nephrotic syndrome in the first year of life: two thirds of cases are caused by mutations in 4 genes (NPHS1, NPHS2, WT1, and LAMB2) Pediatrics 2007 119 04 e907 e919 17371932 10.1542/peds.2006-2164

[2] Reynolds B C Oswald R JA Diagnostic and management challenges in congenital nephrotic syndrome Pediatric Health Med Ther 2019 10 157 167 31908565 10.2147/PHMT.S193684 PMC6930517

[3] Wang J J Mao J H The etiology of congenital nephrotic syndrome: current status and challenges World J Pediatr 2016 12 02 149 158 26961288 10.1007/s12519-016-0009-y

[4] Fofah O Roth P Congenital nephrotic syndrome presenting with cerebral venous thrombosis, hypocalcemia, and seizures in the neonatal period J Perinatol 1997 17 06 492 494 9447540

[5] Horsch S Schaper J Roll C Lesions in congenital nephrotic syndrome J Pediatr 2007 151 02 221 17643784 10.1016/j.jpeds.2007.05.021

[6] Jalanko H Congenital nephrotic syndrome Pediatr Nephrol 2009 24 11 2121 2128 17968594 10.1007/s00467-007-0633-9 PMC2753773

[7] Schwarz H Kohn J L Lipoid nephrosis: A clinical and pathologic study based on fifteen years observation with special reference to prognosis Am J Dis Child 1935 49 03 579 593

[8] Igarashi M Roy S III Stapleton F B Cerebrovascular complications in children with nephrotic syndrome Pediatr Neurol 1988 4 06 362 365 3072964 10.1016/0887-8994(88)90084-7

[9] Konopásek P Piteková B Krejčová V Zieg J Cerebral sinovenous thrombosis in children with nephrotic syndrome: systematic review and one new case Front Pediatr 2023 11 1.207871E6 10.3389/fped.2023.1207871 PMC10484110 37691772

[10] Ranganathan S Pathology of podocytopathies causing nephrotic syndrome in children Front Pediatr 2016 4 32 27066465 10.3389/fped.2016.00032 PMC4814732

[11] Hamasaki Y Hamada R Muramatsu M A cross-sectional nationwide survey of congenital and infantile nephrotic syndrome in Japan BMC Nephrol 2020 21 01 363 32838745 10.1186/s12882-020-02010-5 PMC7446144

[12] Boyer O Schaefer F Haffner D Management of congenital nephrotic syndrome: consensus recommendations of the ERKNet-ESPN Working Group Nat Rev Nephrol 2021 17 04 277 289 33514942 10.1038/s41581-020-00384-1 PMC8128706

[13] Patrakka J Martin P Salonen R Proteinuria and prenatal diagnosis of congenital nephrosis in fetal carriers of nephrin gene mutations Lancet 2002 359 9317 1575 1577 12047969 10.1016/S0140-6736(02)08504-5

